# Sex Differences in Human Myogenesis Following Testosterone Exposure

**DOI:** 10.3390/biology14070855

**Published:** 2025-07-14

**Authors:** Paolo Sgrò, Cristina Antinozzi, Guglielmo Duranti, Ivan Dimauro, Zsolt Radak, Luigi Di Luigi

**Affiliations:** 1 Unit of Endocrinology, Department of Movement, Human and Health Sciences, University of Rome Foro Italico, 00135 Rome, Italy; paolo.sgro@uniroma4.it (P.S.); luigi.diluigi@uniroma4.it (L.D.L.); 2 Unit of Biochemistry and Molecular Biology, Department of Movement, Human and Health Sciences, University of Rome Foro Italico, 00135 Rome, Italy; guglielmo.duranti@uniroma4.it; 3 Unit of Biology and Genetics of Movement, Department of Movement, Human and Health Sciences, University of Rome Foro Italico, 00135 Rome, Italy; ivan.dimauro@uniroma4.it; 4 Research Institute of Sport Science, Hungarian University of Sport Science, 1525 Budapest, Hungary; radak.zsolt@tf.hu

**Keywords:** sex dimorphism, gender medicine, skeletal muscle cells, androgens

## Abstract

Previous research has demonstrated sex-specific differences in muscle cells regarding sex hormone release and steroidogenic enzyme expression after testosterone exposure. The present study aims to elucidate sex-related differences in intracellular processes involved in myogenesis and regeneration. Neonatal 46XX and 46XY human primary skeletal muscle cells were treated with increasing doses of testosterone (0.5, 2, 5, 10, 32, and 100 nM) for 24 h. The molecular pathways involved in muscle metabolism and growth, as well as the release of myokines involved in satellite cell activation, were analyzed using western blot, real-time PCR, and a Luminex assay. The unpaired Student’s *t*-test and one-way ANOVA for repeated measures were used to determine significant variations within and between groups. An increase in the expression and release of MYF6, IGF-I, IGF-II, and CXCL1, as well as a decrease in GM-CSF, IL-9, and IL-12, was observed in 46XX cells. Conversely, testosterone up-regulated GM-CSF and CXCL1 in 46XY cells but did not affect the release of the other myokines. Preferential activation of the MAPK pathway was observed in 46XX cells, while the PI3K/AKT pathway was preferentially activated in 46XY cells. In conclusion, our findings demonstrate differential responses to androgen exposure in 46XX and 46XY cells, resulting in the activation of muscle cell growth and energy metabolic pathways in a sex-specific manner.

## Simple Summary

Sex steroids refer to several hormones such as testosterone, estrogen, and derivatives thereof, which are released mainly by the gonads (testes and ovaries), influencing the formation of primary and secondary sexual characteristics. However, these hormones can also influence the activity of other tissues; in particular, skeletal muscle is one of the main tissue targets of testosterone action. However, the cellular effects of these molecules are different in male and female tissues, although the molecular processes involved in these differences are not fully understood. The aim of this study is to elucidate some of the molecular processes involved in muscle growth and metabolism that may be different in male and female skeletal muscle cells. The importance of characterizing these processes is fundamental to improving clinical approaches and making gender medicine more specific.

## 1. Introduction

Muscle tissue is the most abundant tissue in the human body. It is the target of hormones such as insulin, growth factors, and sex hormones, which regulate muscle metabolism, strength, mass, cell growth, and proliferation [1,2,3]. Sex hormones have been shown to exert different effects on muscle cells function, depending on factors such as gender, sex, muscle cell types, and muscle anatomical position [4,5,6,7,8]. However, the role of sex hormones in activating intracellular signaling pathways in muscles remains an area of ongoing research. This is because different studies have yielded conflicting results regarding the effects of androgens and estrogens on signal transduction and the modulation of genes and protein targets [4,5,6,7]. In particular, studies examining the correlation between estrogen and myogenesis remain scarce. Research conducted on ovariectomized female mice demonstrated that the absence of estrogenic hormones resulted in muscle atrophy, reduced strength, a transition toward fast-twitch fibers, and impaired satellite cell function [7]. Despite these findings, a meta-analysis of postmenopausal women reported no significant benefit of estrogen replacement therapy (ERT) on muscle mass, likely due to confounding factors such as reduced physical activity and dietary changes [9]. In men, ERT has only been the subject of sporadic research in cases of rare aromatase deficiency, although the decline in estradiol with advancing age has been demonstrated to have an impact on skeletal health [9]. Further discrepancies have been observed between transgender men and women undergoing hormone therapy with estrogen and/or testosterone. These discrepancies concern respiratory efficiency during physical activity and the mental approach to exercise [4,5,6]. However, it is evident that further research is required to define the role of sex hormones in muscle and evaluate their role in both sexes.

Previously sex chromosome-related differences in the expression of steroidogenic enzymes and hormone biosynthesis in human primary skeletal muscle cells following testosterone treatment have been demonstrated. Specifically, distinct steroidogenesis, sex steroids receptor expression, and different hormonal profiles based on the presence of sex chromosomes have been found in 46XX and 46XY human skeletal muscle cells [10]. Furthermore, it was hypothesized that this difference in hormone homeostasis influences several aspects of energy management, including mitochondrial function, substrate utilization, insulin sensitivity, and the balance between anabolic and catabolic processes, which, in turn, affect the expression and release of key myokines involved in metabolic regulation [11,12,13,14].

In this study, we examined the effects of testosterone (T) treatment on 46XX and 46XY cells at increasing doses (0.5, 2, 5, 10, 32, and 100 nmol/L), simulating normo-, hypo-, and hyper-androgenic conditions [15]. We analyzed the activation of molecular pathways involved in muscle metabolism and growth, specifically, those associated with sex hormone stimulation. In particular, we studied the activation of mitogen-activated protein kinase (ERK/MAPK) 1/2 and protein kinase B (AKT), focusing on their roles in cell proliferation and survival, as well as myogenic factor (MYF)-6 gene expression. We also examined the release of factors involved in satellite cell activation, as well as insulin-like growth factors (IGF) I and II, granulocyte macrophage colony-stimulating factor (GM-CSF), CXC motif chemokine ligand (CXCL) 1, interleukin (IL)9, and interleukin (IL)12, all of which are involved in cell proliferation, growth, and differentiation.

This research provides notable indications that cell responses to testosterone are not consistent but are influenced by genetic sex, thus emphasizing the biological significance of sex-related differences at the cellular level. This study makes a valuable contribution to our understanding of sexual dimorphism in skeletal muscle biology, highlighting the different anabolic and metabolic effects of testosterone on muscle.

## 2. Materials and Methods

### 2.1. Cell Cultures and Treatments

Human primary skeletal muscle cells isolated from vastus lateralis muscle were purchased from ATCC (PCS-950-010TM, Manassas, VA, USA) and LONZA (LOCC2580, Basel, Switzerland) and were cultured and treated as previously described [10]. Cells isolated from neonatal donors (0–1 months of age) were cultured in Cell Basal Medium (ATCC PCS-500-030) plus one Primary Skeletal Cell Muscle Growth Kit (ATCC PCS-950-040) in an atmosphere of 5% CO_2_ at 37 °C. Once the cells had reached about 70–80% confluence, the growth medium was replaced with fresh medium or plated to perform each experimental analysis. Briefly, cells were plated and treated for 24 h with increasing doses of testosterone (T) (Sigma Aldrich, St. Louis, MO, USA). T was administered at concentrations of 0, 0.5, 2, 5, 10, 32, and 100 nmol/L (or nM), representing a range of physiological and non-physiological serum total testosterone levels. Physiological concentrations were defined as 0.5–2 nmol/L for females and 10–32 nmol/L for males, while non-physiological levels were considered as >2 nmol/L for females and <10 or >32 nmol/L for males [16]. For experiments concerning mRNA expression and myokines release, five different lots of primary cells from two different donors were used, while for western blot analysis, three different lots of primary cells from two different donors were used. Before manipulation, both 46XX and 46XY cells had never been exposed to sexual steroid hormone environments causing the transformation of a sexually immature child into a sexually mature adult. Each experiment was performed in triplicate in serum free conditions and without phenol red.

### 2.2. RNA Extraction, Reverse Transcription, and Real-Time Quantitative PCR

Total RNA was obtained from ≈3.5 × 104 cells using TRIZOL reagent according to the manufacturer’s instructions. Treatment with DNAse enzyme was performed to remove genomic DNA contamination. cDNA was obtained by reverse transcription of 500 ng of total RNA. RT-qPCRs were performed as previous study [10]. Fluorescence intensities were analyzed using the manufacturer’s software (7500 Software v2.05), and relative amounts were evaluated using the 2^−∆Ct^ method and normalized for β-actin. Data are expressed as 2^−∆Ct^ or 2^−∆∆Ct^ (arbitrary unit). Sequences of primers are shown in Table 1.

### 2.3. Protein Expression Analysis

Protein expression analysis was performed as previously described [17]. Briefly, after each treatment, 46XY and 46XX cells were lysed in RIPA buffer (150 mM NaCl, 50 mM tris-HCl pH8, 1 mM EDTA, 1% NP40, 0.25% sodium deoxycholate, 0.1% SDS, water to volume), supplemented with protease and phosphatase inhibitor cocktails (Sigma-Aldrich). An equal amount of protein (20–30 µg) was then resolved in SDS-polyacrylamide (BIO-RAD) gels (10–12%) and transferred onto nitrocellulose membranes (Amersham). Membrane blocking with Bovine Serum Albumine (BSA) 5% in Tween Tris-buffered saline (TTBS) was performed. Thereafter, membranes were incubated with primary antibodies appropriately diluted in TTBS (for anti-p-ERK, anti-p-AKT, anti-AKT, anti-ERK 1:1000), followed by peroxidase-conjugated secondary IgG (1:10,000). Primary and secondary antibodies were purchased from Santa Cruz Biotechnology (Santa Cruz, CA, USA). Proteins were revealed by an enhanced chemiluminescence system (ECL plus; Millipore, Burlington, MA, USA). Image acquisitions were performed with Image Quant Las 4000 software (GE Healthcare, Chicago, IL, USA), and densitometric analysis was performed with Quantity One^®^ software 4.6.6 (Bio-Rad laboratories Inc., Hercules, CA, USA).

### 2.4. Cytokines Assay

Female or 46XX and male or 46XY skeletal muscle cells were plated at 2 × 104 cells/mL in 96-well tissue culture plates and exposed to testosterone as describes in the cell culture and treatment section. Supernatants were assayed for IGF-I, IGF-II, GM-CFS, IL9, IL12p40, CXCL1 by magnetic bead-based multiplex assay according to the manufacturer’s protocol. As previously described [18], data acquisition was performed using a Bio-Plex 200 System™ (Bio-Rad Laboratories, Inc., Hercules, CA, USA). Data analysis was performed by Bio-Plex Manager™ 6.0 software (Bio-Rad Laboratories, Inc., Hercules, CA, USA). Quality control pools of low, normal, and high concentrations for all parameters were included in each assay. Data are expressed as pg/mL. Cells supernatants were run in triplicate.

### 2.5. Statistical Analysis

All data are expressed as means ± standard deviation (SD) from three independent repeats, each performed in triplicate. Prior to applying parametric tests, data distribution was assessed for normality using the Shapiro–Wilk test, and homogeneity of variances was evaluated using Levene’s test to ensure that the assumptions of parametric statistics were met. An unpaired two-tailed Student’s *t*-test was used to assess significant differences within groups. For comparisons among multiple groups, a one-way ANOVA for repeated measures followed by Bonferroni post hoc correction was applied. A *p*-value < 0.05 was considered statistically significant. Although the sample size was limited to three independent experiments, the use of technical triplicates and consistent effect sizes across experiments support the reliability of the findings. Statistical analyses were performed using GraphPad Prism version 9 (GraphPad Software, Boston, MA, USA) and SPSS software version 17.0 (SPSS Inc., Chicago, IL, USA). Given the exploratory nature of the study and the limited availability of clinical samples, a reduced number of patient-derived specimens was used; however, the sample size was sufficient to detect consistent and biologically relevant effects.

## 3. Results

### 3.1. Testosterone Differently Modulates Myogenesis in Male and Female Human Skeletal Muscle Cells

**Figure 1:**
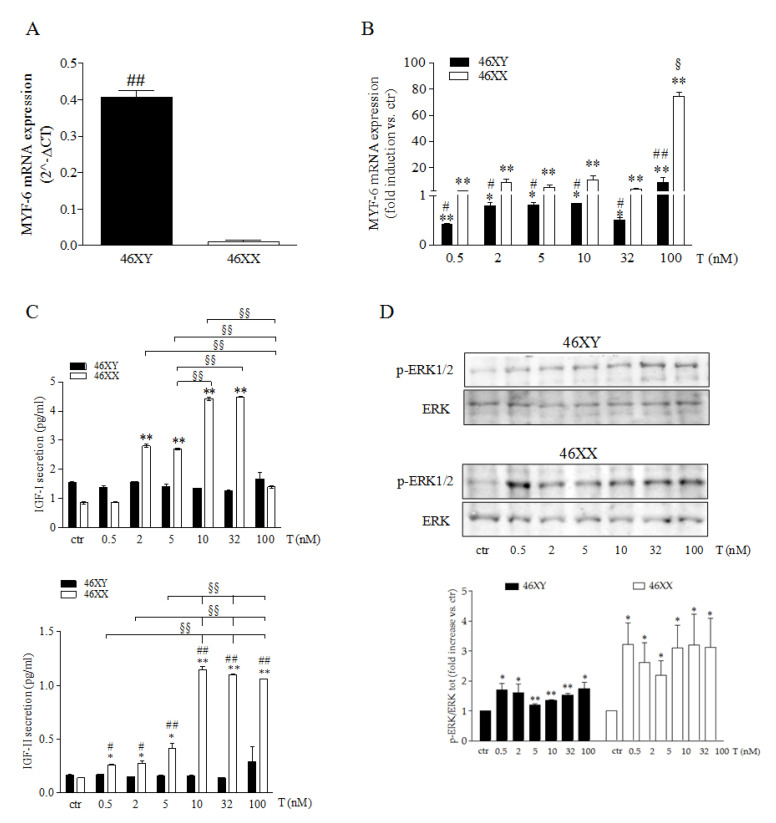
Myogenic process control in male and female muscle cells. (**A**) Basal level of MYF-6 mRNA in 46 XY and 46XX cells. Data are expressed as 2^−ΔCT^ ± SD. (**B**) Modulation of MYF-6 mRNA in 46 XY (black columns) and 46XX (white columns) cells after T exposure. Data are expressed as fold induction vs. control (ctr) taken as 1. (**C**) IGF-I and IGF-II analysis in cell culture medium after T exposure. Data are expressed as pg/mL. (**D**) Representative western blot of *p*-ERK1/2 and total ERK1/2 46XY (upper panel) and 46XX (lower panel) cells. Data are expressed as folding increase versus control taken as 1. (**A**–**D**) Histograms represent densitometric analysis of three independent experiments. Black columns represent 46XY cells, whereas white columns represent 46XX cells. Statistical significance was determined by an ANOVA with Bonferroni’s post hoc test. * *p* < 0.05, ** *p* < 0.01 vs. ctr; # *p* < 0.05, ## *p* < 0.01 46XY vs. 46XX cells; § *p* < 0.05, §§ *p* < 0.01 vs. other T concentrations. (**A**–**C**) Data were performed three times with five different preparations.

We tested whether sex dimorphism in vitro could affect skeletal muscle cells muscle growth and development in relation to T treatment at the different doses analyzed. As shown in Figure 1, we observed at the basal level that 46XX and 46XY cells significantly differed for MYF-6 mRNA (*p* = 2^−5^), with an amount of 0.4 ± 0.0 in 46XY cells (Figure 1A, black columns) in comparison to the undetectable levels observed in 46XX cells (Figure 1A white columns. T exposure modulated the expression of MYF-6 mRNA distinctly in 46 XX (Figure 1B white columns) and 46XY cells (Figure 1B black columns). In 46XX cells, T significantly increased MYF-6 expression by 2.0 ± 0.4-fold at 0.5 nM (*p* = 0.030), by 8.7 ± 2.6-fold at 2 nM (*p* = 0.020), by 4.9 ± 1.6-fold at 5 nM (*p* = 0.038), by 10.7 ± 3.1-fold at 10 nM (*p* = 0.017), by 3.6 ± 0.9-fold at 32 nM (*p* = 0.018), and by 74.6 ± 3.5-fold at 100 nM (*p* = 1.4^−05^) (Figure 1B white columns). In 46XY cells, T significantly increased MYF-6 mRNA by 8.8 ± 4.0-fold at 100 nM (*p* = 0.010) and decreased MYF-6 mRNA by 0.6 ± 0.0-fold at 0.5 nM (*p* = 7.1^−05^), by 0.2 ± 0.1-fold at 2 nM (*p* = 0.023), by 0.2 ± 0.0-fold at 5 nM *p* = 0.005), by 0.2 ± 0.0-fold at 10 nM (*p* = 0.030), and by 0.5 ± 0.0-fold at 32 nM (*p* = 0.011) (Figure 1B black columns).

Next, we analyzed the release of the growth factor IGF-I and IGF-II (Figure 1C). In 46XY, we did not observe modulations related to T exposure (black columns), whereas in 46XX cells, we observed an increase in IGF-I of 2.8 ± 0.1 and 2.7 ± 0.0 with T 2 and 5 nM (*p* = 4.8^−6^ and *p* = 1.5^−6^), an increase of 4.4 ± 0.0 and 4.5 ± 0.0 with T 10 and 32 nM (white columns) (*p* = 3.2^−7^ and *p* = 6.2^−8^), and an increase in IGF-II of 1.9 ± 0.0 fold, with T 2 and 5 nM (*p* = 0.016 and *p* = 0.022), 3.0 ± 0.0 fold with T 5 nM (*p* = 0.41), 8.0 ± 0.1 fold with T 10 nM, 32 nM, and 100 nM (*p* = 5.5^−5^ and *p* = 1.7^−5^). After that, we analyzed the activation of ERK1/2. In both 46XX and in 46XY cells, T induced a significantly increased of ERK phosphorylation at any dose utilized, with an greater increase in 46XX cells than in 46XY cells (Figure 1D and Figure S2) (*p* = 0.02 after T 0.5 nM, *p* = 0.04 after T 2 nM, *p* = 0.03 after T 5 nM, *p* = 0.02 after T 10 nM, *p* = 0.04 after T 32 nM, and *p* = 0.04 after T 100 nM in 46XX cells; *p* = 0.02 after T 0.5 nM, *p* = 0.04 after T 2 nM, *p* = 0.005 after T 5 nM, *p* = 2.3^−06^ after T 10 nM, *p* = 0.0005 after T 32nM, and *p* = 0.02 after T 100 nM in 46XY cells).

### 3.2. Testosterone Differently Modulates Myokine Release in Male and Female Human Skeletal Muscle Cells

**Figure 2:**
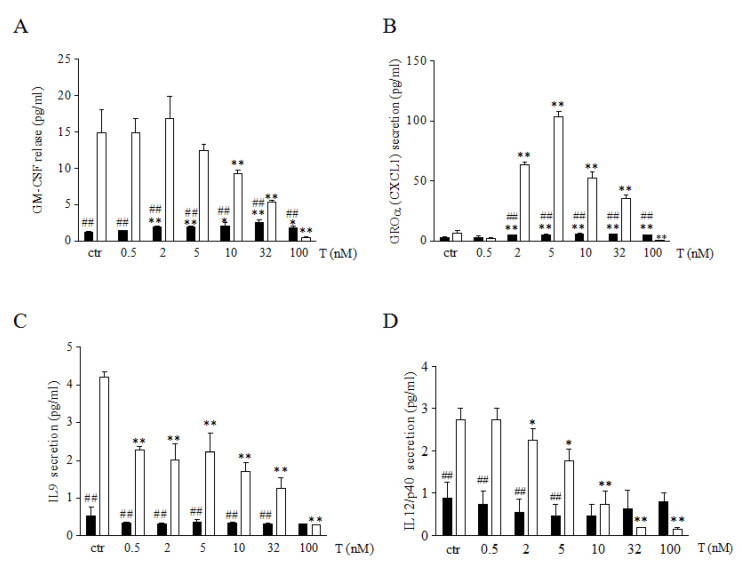
Myokine analysis in cell culture medium. Cells were analyzed for GM-CSF (**A**), GROα (**B**), IL9 (**C**) and IL12p40 (**D**) release after different concentrations of T exposure. Data are expressed as pg/mL ± SD. Black columns represent 46XY cells, whereas white columns represent 46XX cells. * *p* < 0.05, ** *p* < 0.01 vs. relative control within group (ctr); ## *p* < 0.01 vs. corresponding treatment between groups (46XY vs. 46XX cells). Statistical significance was determined by an ANOVA with Bonferroni’s post hoc test.

We analyzed the release of GM-CFS, IL9, IL12, and CXCL1 myokines implicated in energy metabolism, cell growth, and proliferation. As shown in Figure 2 (panel A–D), 46XX cells, at basal condition, showed greater amounts of all myokines analyzed in comparison to 46XY cells, (respectively: for GM-CFS, 14.9 ± 3.2 vs. 1.2 ± 0.2, *p* = 0.0106 for CXCL1, 4.3 ± 2.5 vs. 3.2 ± 0.2, *p* = 0.07 (NS); for IL9, 4.2 ± 0.1 vs. 0.8 ± 0.0, ## *p* = 0.0005; for IL12/p40, 2.7 ± 0.3 vs. 1.3 ± 0.0, ## *p* = 0.0007;). However, in 46XY cells, T induced, a statistical significant increase of GM-CFS and CXCL1 (Figure 2A, B, black columns) after 2, 5, 10, 32 and 100 nM of treatment was observed (for GM-CFS: *p* = 0.006 after T 2 nM, *p* = 0.009 after T 5 nM, *p* = 0.04 after T 10 nM, *p* = 0.005 after T 32 nM and *p* = 0.05 after T 100 nM; for CXCL1: *p* = 0.001 after T 2 nM, *p* = 0.001 after T 5 nM, *p* = 0.014 after T 10 nM, *p* = 0.05 after T 32 nM and *p* = 0.05 after T 100 nM). Conversely, in 46XX cells, T significantly decreased GM-CFS secretion after 10, 32, and 100nM of treatment (Figure 2A, white columns) (*p* = 0.07 after T 10 nM, *p* = 0.05 after T 32 nM, and *p* = 0.05 after T 100 nM), IL9 (Figure 2C, white columns) (*p* = 0.001 after T 0.5 nM, *p* = 0.004 after T 2 nM, *p* = 0.008 after T 5 nM, *p* = 0.0003 after T 10 nM, *p* = 0.05 after T 32 nM, and *p* = 0.05 after T 100 nM in 46XY cells) and IL12/p40 after 2, 5, 10, 32 and 100nM of treatment (*p* = 0.03 after T 2 nM, *p* = 0.04 after T 5 nM, *p* = 0.05 after T 32 nM, and *p* = 0.05 after T 100 nM in 46XY cells) (Figure 2D, white columns). In contrast, the analysis of CXCL1 demonstrated in 46XX cells that T treatment induced a high increase of chemokine secretion after 2, 5, 10, and 32 nM of treatment (*p* = 0.004 after T 2 nM, *p* = 0.0003 after T 5 nM, *p* = 0.0003 after T 10 nM and *p* = 0.0002 after T 32 nM) and a statistical significant decrease after 100 nM of treatment (*p* = 0.008) (Figure 2B, white columns).

### 3.3. Testosterone Differently Modulates PI3K/AKT Activation Release in Male and Female Human Skeletal Muscle Cells

**Figure 3:**
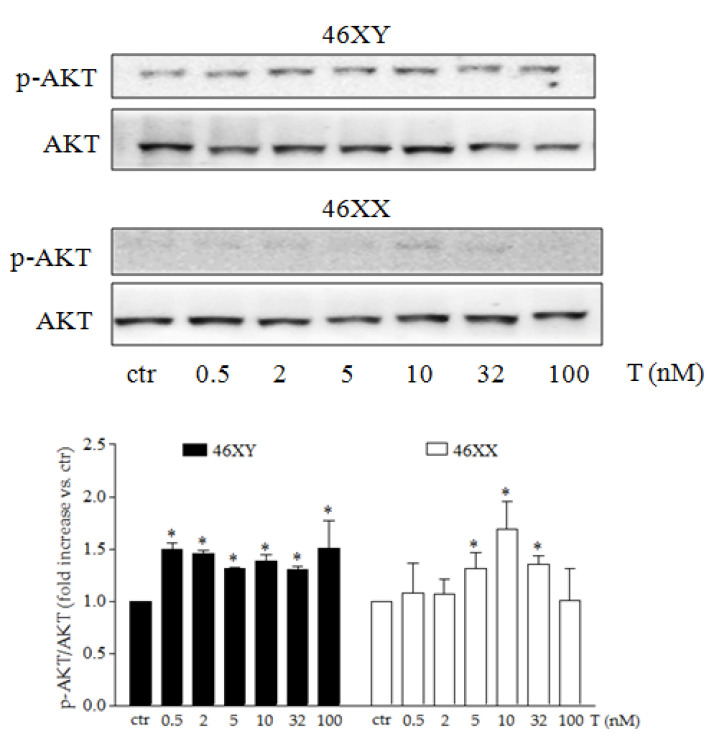
AKT activation after T exposure. Representative western blot of *p*-AKT and total AKT in 46XY and 46XX cells. Histograms below represent densitometric analysis of three independent experiments. Black columns represent 46XY cells, whereas white columns represent 46XX cells. Data are expressed as fold increase versus control taken as 1. The dotted red line indicates control levels. Statistical significance was determined by an ANOVA with Bonferroni’s post hoc test. * *p* < 0.05, vs. ctr.

Finally, we analyzed the activation of PI3K/AKT, one of the most important signal transduction pathways involved in cell energy regulation. As shown in Figure 3 and Figure S2, 46XY strongly activated AKT already at lowest T concentration and maintained the phosphorylation at each dose of treatment (Figure 3 black columns). In contrast, in 46XX, we observed an almost undetectable level of *p*-AKT, while a significantly increase was observed only after testosterone exposure at 5, 10, and 32 nM (Figure 3 white columns) (*p* = 0.39 after T 0.5 nM, *p* = 0.33 after T 2 nM, *p* = 0.049 after T 5 nM, *p* = 0.03 after T 10 nM, *p* = 0.007 after T 32 nM, and *p* = 0.49 after T 100 nM in 46XX cells; *p* = 0.04 after T 0.5 nM, *p* = 0.03 after T 2 nM, *p* = 0.02 after T 5 nM, *p* = 0.0013 after T 10 nM, *p* = 0.03 after T 32 nM, and *p* = 0.048 after T 100 nM in 46XY cells).

## 4. Discussion

This in vitro study highlights a dimorphism in testosterone’s effects on 46XY and 46XX human skeletal muscle cells, specifically regarding muscle metabolism and cell biogenesis. In 46XX cells, testosterone primarily activated myogenesis-related processes, whereas in 46XY cells, it activated energy metabolism-related pathways.

Testosterone is crucial for muscle homeostasis, regulating anabolic and catabolic mechanisms. It exerts both genomic and non-genomic actions, influencing fiber distribution, increasing lean mass and strength, and decreasing fat mass in both genders [19,20,21]. Furthermore, testosterone promotes muscle growth and regeneration through androgen receptor interaction [17,22,23,24,25], while at the molecular level, it affects proliferation, differentiation, and strength production by binding to the androgen receptor and modulating satellite cell number, myofiber nuclei, and the expression of transcription factors such as myostatin, myogenic factor, and myogenin, which are involved in muscle development and regeneration [26,27].

Early studies of sex differences concerning the action of testosterone in muscle tissue were conducted on male and female rats and showed that androgen exposure altered myosin heavy chain expression only in females, with no effect in males [28]. In addition, some animal models suggest that sex hormones, including testosterone and estradiol, may have sex-specific effects on muscle mass and cell regeneration [29]. However, further research is needed to investigate the sexual dimorphism in muscle tissue exposed to testosterone and to investigate the molecular mechanisms involved in muscle metabolism.

Previously, we demonstrated a sex difference in 46XX and 46XY skeletal muscle cells in term of steroidogenesis in response to testosterone at different concentrations [10]. However, other aspects of cell metabolism, such as myogenesis and energy metabolism, remain unexplored.

In this study, we observed that 46XX muscle cells increased the expression of MYF-6, activation of *p*-ERK1/2, and the release of CXCL1 and IGF-I, and IGF-II, all of which are molecules involved in satellite cell activation. In contrast, 46XY cells showed preferential induction of cell regeneration (GM-CFS) and activation of energy metabolism (pAKT activation).

Initial observations revealed significant differences in MYF-6 expression between male and female cells. In particular, 46XY cells exhibited higher levels of MYF-6 mRNA than 46XX cells under basal conditions. However, compared to the untreated controls, 46XX cells demonstrated a significant increase in MYF-6 mRNA expression, whereas 46XY cells exhibited a notable decrease (Figure 1A,B). MYF-6 is a myogenic factor that is essential for muscle myogenesis and the inhibition of cell differentiation. Myf6 knockout mice exhibited a gradual reduction in their stem cell population and a tendency to exit quiescence [30]. MYF-6 exerts its effects by regulating the expression of a wide spectrum of myokines and growth factors, which are probably activated via the mitogen-activated protein kinase (MAPK) signaling pathway [30].

Consistent with these findings, we observed an increase in IGF-I, IGF-II, and CXCL1 release in 46XX, accompanied by a notable elevation in ERK1/2 phosphorylation following testosterone exposure (Figure 1C,D and Figure 2B). This observation suggests a more extensive cellular response involving both anabolic and proliferative signaling (Figure S1), which is likely to contribute to tissue remodeling and regeneration. It is important to note that the expression of IGF-I was induced even at lower testosterone doses, whereas IGF-II required higher concentrations for significative upregulation. This differential sensitivity may be indicative of distinct transcriptional regulation mechanisms, with the IGF-I promoter demonstrating a heightened response to androgenic stimulation or early coactivators. Conversely, IGF-II, which is typically active during development and regeneration, may necessitate more sustained or combined signals, potentially including epigenetic modulation or autocrine feedback [31]. Finally, activation of the ERK1/2 signaling pathway may enhance the self-renewal of satellite cells, induce the downstream activation of the c-JUN transcription factor, and increase the expression of Pax7 [32,33,34]. In 46XY cells, we observed a lack of significant ERK phosphorylation, as well as an absence of significative increases in IGF-I or IGF-II release into the culture medium. These findings suggest that, in this cellular context, testosterone may directly activate the AKT/mTOR axis via the androgen receptor (AR), thus bypassing the need for secreted growth factors, such as insulin-like growth factors (IGFs) or growth hormone (GH) [35,36]. The hypothesis is that insulin-like growth factor (IGF) is synthesized intracellularly but not secreted, or that its secretion is less sensitive to testosterone and takes much longer to be observed. To support these hypotheses, it has previously been demonstrated that 46XX cells activate AR translocation at lower testosterone doses and that the same effects start to be observed in 46XY cells at the highest testosterone concentrations [10].

Whereas IGF-I and IGF-II play a pivotal role in preserving muscle mass through the stimulation and differentiation of satellite cells [37,38], CXCL1 has been extensively investigated in cancer models, where it has been shown to be implicated in the proliferation and migration of both cancer cells and tumor-associated cells [39]. CXCL1 recruits immune cells, particularly neutrophils and myeloid-derived suppressor cells, and induces angiogenesis [39]. However, beyond its pathological role, Masuda and colleagues demonstrated that CXCL1 is essential for maintaining skeletal muscle homeostasis, promoting myogenesis [40]. Notably, Hu and colleagues [41] observed an inverse correlation between CXCL1 and CCL2 in serum samples from 24 patients, suggesting that these cytokines may serve as potential novel predictors of early bone loss and be clinically relevant for the diagnosis and prevention of osteoporosis. It was observed that CXCL1 levels increased more in 46XX cells than in male cells. It is likely that, in addition to its role in muscle regeneration, 46XX cells secrete CXCL1 to stimulate a paracrine mechanism crucial for maintaining skeletal muscle homeostasis and enhancing the anti-osteoporotic effects of estradiol.

In this context, our prior findings demonstrated that 46XX cells preferentially converted testosterone to estradiol, leading to a significant reduction in the release of IL6 and IL8 myokines. In contrast, male cells exhibited significantly higher levels of these myokines [14]. In addition to their established role as “energy sensors” during muscle contraction and exercise [39], myokines are crucial in bone resorption and the stimulation of CFU-granulocyte macrophage (CFU-GM), as well as IL12 and IL9, which, in turn, promote bone resorption. Further analysis showed a marked decrease in GM-CSF, IL12, and IL9 release in relation to testosterone concentration, while testosterone levels were either slightly increased or unregulated in 46XX cells (Figure 2C,D). Finally, analysis of AKT activation revealed a significant increase in phosphorylation in 46XY cells (Figure 3). This supports previous observations indicating an elevation in IL6 and IL8 levels, reinforces the idea that testosterone serves as both an energy modulator and anabolic sensor, particularly in 46XY muscle cells, and confirms the sexual dimorphism in the structure, function, and energy metabolism in skeletal muscle [42].

While this study yielded new insights, its findings are constrained by several limitations. The first limitation is the number of donors. Given the exploratory nature of the study and the limited availability of clinical samples, a reduced number of patient-derived specimens was used; however, the sample size was sufficient to detect consistent and biologically relevant effects. Nevertheless, to strengthen the investigation, it is recommended that three additional replicates using samples from different donors be performed. Moreover, no evidence was found to support the hypothesis of sexual dimorphism in myotubes. It is established that the anabolic and metabolic actions of testosterone are distinct in myoblasts and myocytes and change in relation to age and health status [43,44]. It is therefore evident that further investigation concerning differences in the responses observed in the two experimental conditions may provide further insights into the observed sexual dimorphism. In this context, experiments under differentiated conditions are underway to analyze the molecular mechanisms responsible for this dimorphism. Finally, the use of receptor antagonists, agonists, and/or inhibitors will be necessary to establish which pathway is favored over another.

## 5. Conclusions

**Figure 4:**
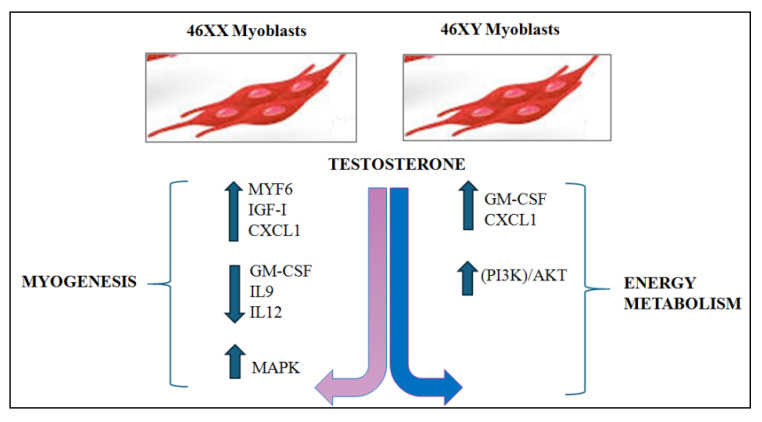
Hypothesized schematic of the differential cellular pathway activations in 46XX and 46XY cells exposed to testosterone. It has been demonstrated that testosterone treatment in 46XX cells results in the modulation of a range of myokines and molecular factors that are predominantly implicated in myogenic processes. Conversely, in 46XY cells, the treatment predominantly activated molecular processes associated with energy metabolism. The blue and pink arrows represent respectively the male and female via activated.

In conclusion, the present study demonstrated that testosterone activates distinct intracellular pathways in 46XX and 46XY skeletal muscle cells, influenced by the presence of sex chromosomes (Figure 4). The findings of this study provide significant evidence that cellular responses to testosterone are not uniform but are shaped by genetic sex, thereby underscoring the biological relevance of sex-based differences at the cellular level. The present study makes a significant contribution to the understanding of sexual dimorphism in skeletal muscle biology by highlighting the differential anabolic and metabolic actions of testosterone in muscle. These insights establish the foundation for a more in-depth understanding of hormone signaling in muscle tissue, with potential implications for both basic research and clinical applications. It is important to note that this work reinforces the necessity of considering sex not a confounding variable but a fundamental experimental factor in in vitro models and will be useful in terms of interpreting data. It is imperative to acknowledge and account for sex-based cellular differences to ensure the accuracy and reproducibility of results, thereby enhancing the translational potential of preclinical studies. In this context, the study can be viewed as a significant advancement in the field of gender-sensitive approaches in muscle research and precision medicine.

## Figures and Tables

**Table 1:** Sequences of primers for RT-PCR analysis.

Gene Name	Forward 5′–3′	Reverse 5′–3′
MYF-6	GAAGATCCCACCGACCCTCCTGGC	GAGGCTAGACCTAAGCCACTCGCA
Β-ACTIN	AAC CTGAACCCCAAGGCC	AGCCTGGATAGCAACGTACA

## Data Availability

The data presented in this study can be obtained on request from the corresponding author.

## Supplementary Materials

The following supporting information can be downloaded at https://www.mdpi.com/article/10.3390/biology14070855/s1, Figure S1: The effect of different concentrations of testosterone on the proliferation rate; Figure S2: Representative western blot images.
