# Supplementary material for: Sex Differences in Human Myogenesis Following Testosterone Exposure
Source: Biology (Basel). 2025 Jul 14;14(7):855. doi: 10.3390/biology14070855 (PMC12293079; doi:10.3390/biology14070855)
Supplement: Supplementary file 1 [file biology-14-00855-s001.zip › Figure Supplementary.pdf]

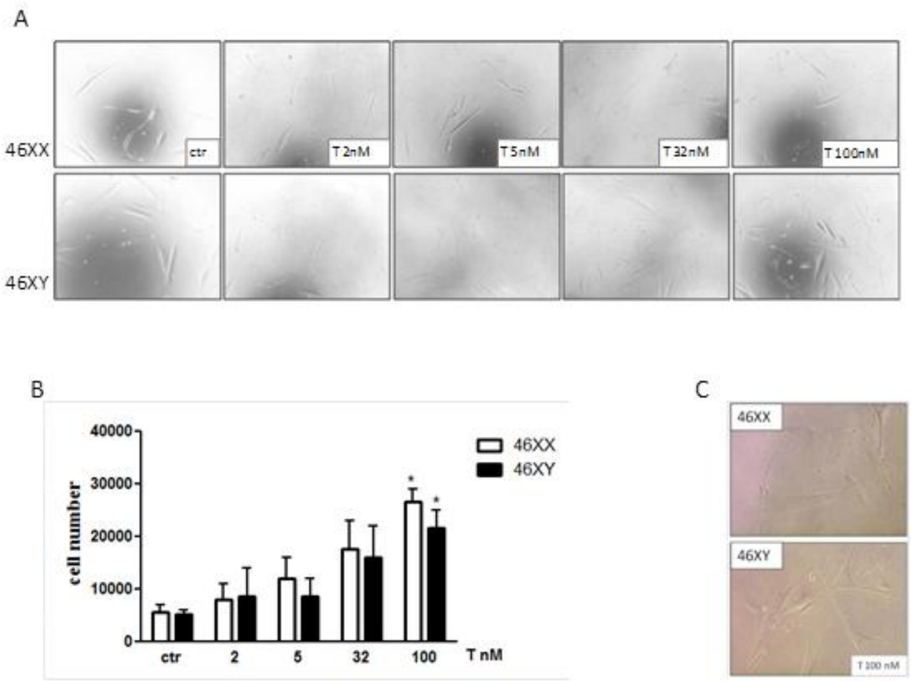

Figure S1. **The effect of different concentrations of testosterone on the proliferation rate.** Morphological analysis (A) of male (46XY, black columns) and female (46XX white columns) cells treated with T at 2, 5, 32 and 100 nM. Histograms (B) represent cell number after 24h of T at 2, 5, 32 and 100 nM (**p<0.05 vs*. ctr). C) Shape analysis of male (46XY) and female (46XX) cells after 24h of T 100nM.

A

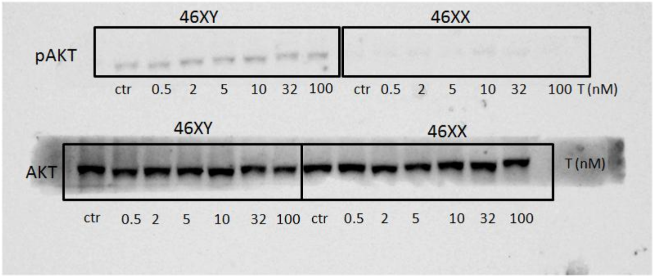

B

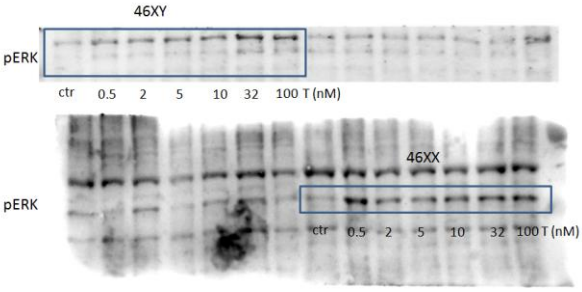

C

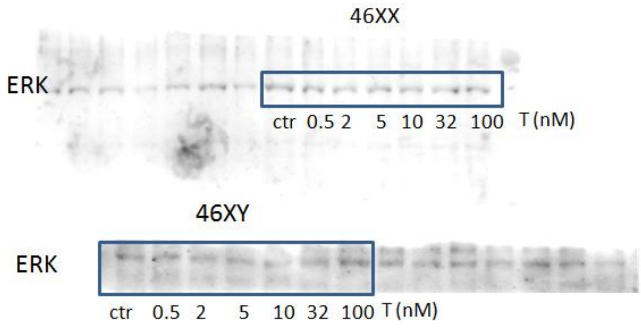

Figure S2. **Representative western blot images**. Original western blot images of p-AKT and total AKT (panel A), *p*-ERK1/2 (panel B) and total ERK1/2 (panel C) in 46XY and 46XX cells. Cells were treated with T at 0.5, 2, 5, 10, 32 and 100 nm for 10 minutes and analyzed as described in the main text.
