# Supplementary material for: Sex Differences in Human Myogenesis Following Testosterone Exposure
Source: Biology (Basel). 2025 Jul 14;14(7):855. doi: 10.3390/biology14070855 (PMC12293079; doi:10.3390/biology14070855)
Supplement: Supplementary file 1 [file biology-14-00855-s001.zip › western blot.pdf]

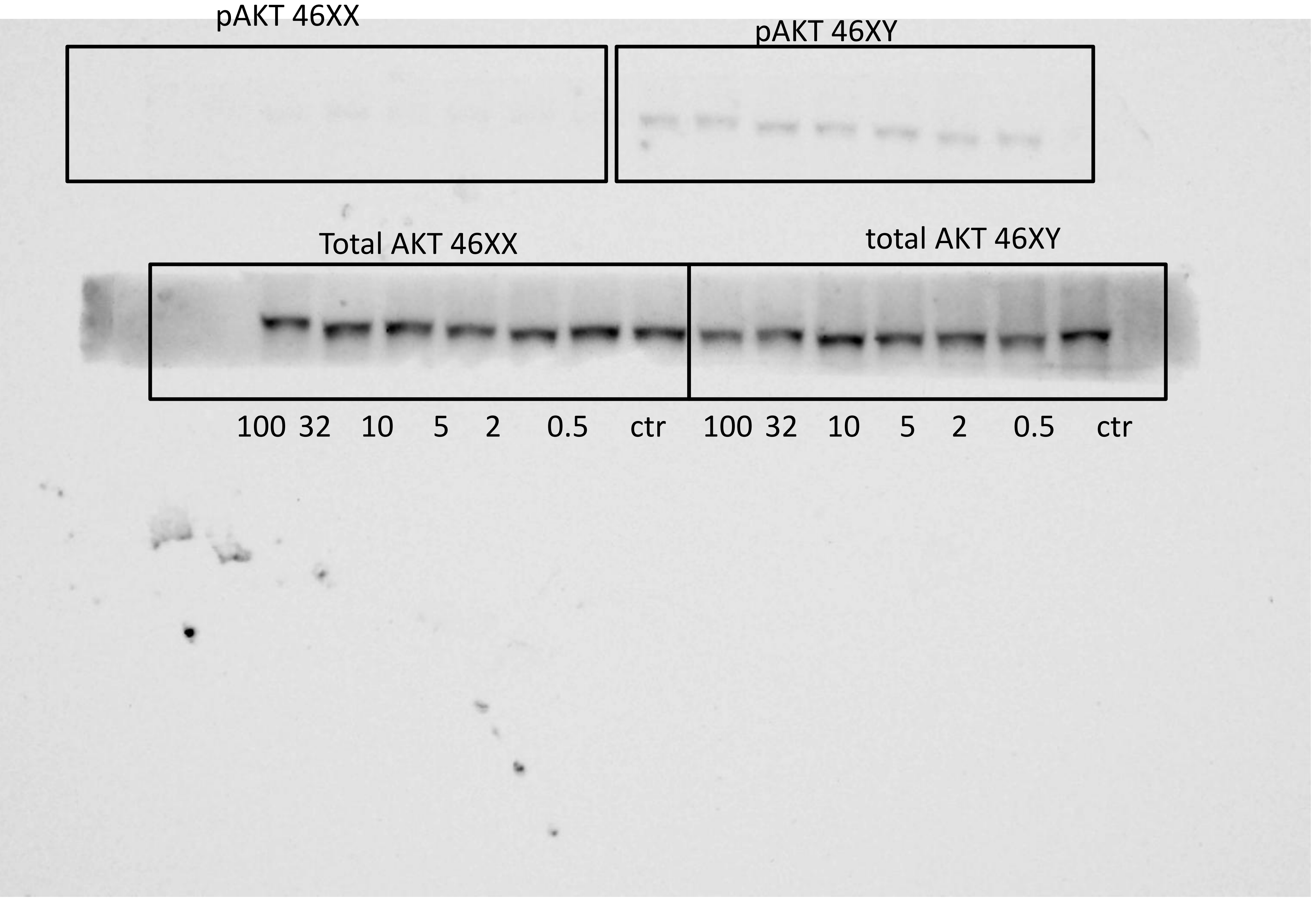
pAKT 46XX
pAKT 46XY
Total AKT 46XX
total AKT 46XY
100 32 10 5 2 0.5 ctr
100 32 10 5 2 0.5 ctr

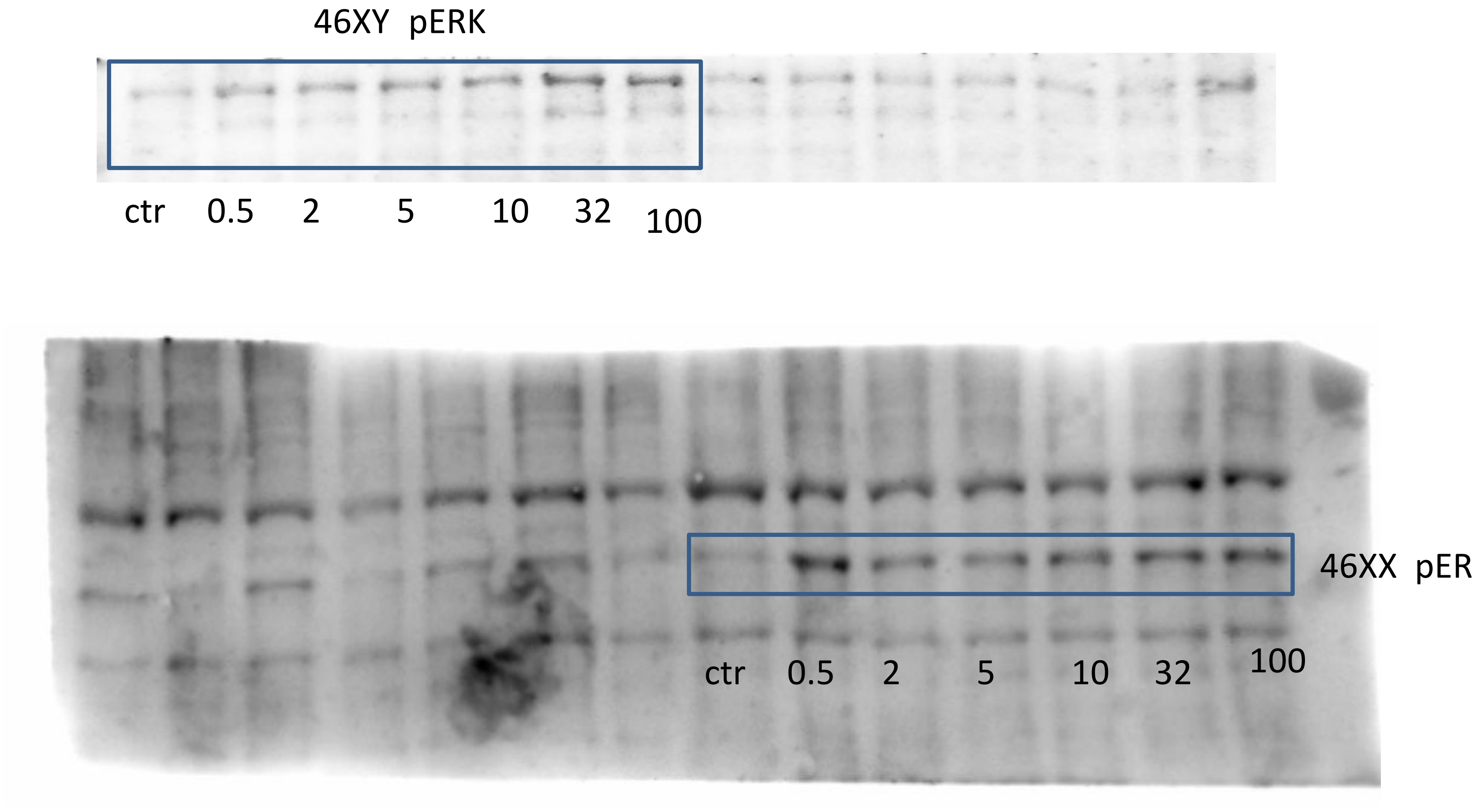
46XY pERK
ctr
0.5
2
5
10
32
100
46XX pER
ctr
0.5
2
5
10
32
100

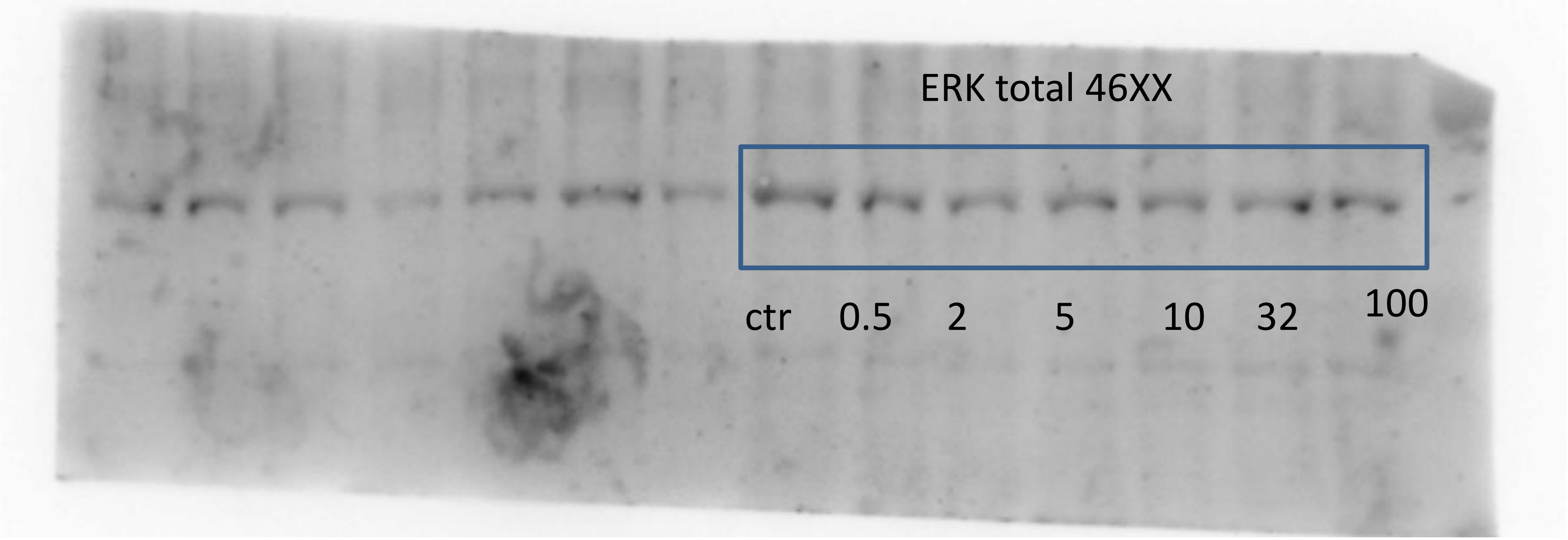
ERK total 46XX
ctr
0.5
2
5
10
32
100

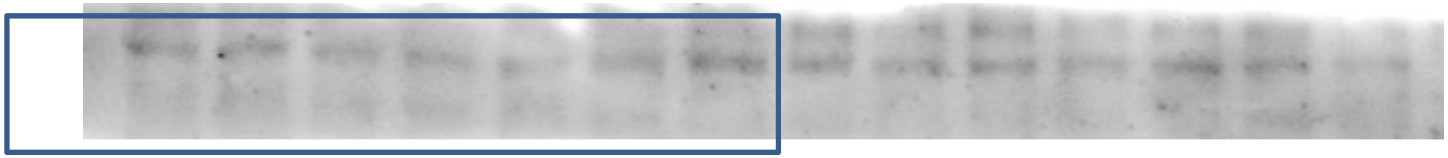
ERK total 46XY
100
32
10
5
2
0.5
ctr
